# The effect of tai chi and Qigong exercise on depression and anxiety of individuals with substance use disorders: a systematic review and meta-analysis

**DOI:** 10.1186/s12906-020-02967-8

**Published:** 2020-05-29

**Authors:** Fang Liu, Jiabao Cui, Xuan Liu, Kevin W. Chen, Xiaorong Chen, Ru Li

**Affiliations:** 1 Faculty of Physical Education, Shenzhen University, Shenzhen, 518060 China; 2 Center for Integrative Medicine, University of Maryland School of Medicine, Baltimore, USA

**Keywords:** Qigong, Tai ji, Depressive disorder, Anxiety, Substance-related disorders, Review

## Abstract

**Background:**

Previous studies have acknowledged Tai Chi and Qigong exercise could be potential effective treatments for reducing depression and anxiety in both healthy and clinical populations. However, there is a scarcity of systematic reviews summarizing the clinical evidence conducted among individuals with substance use disorders. This study tries to fill up this gap.

**Methods:**

A systematic search using Medline, EMbase, PsychINFO, Eric, SPORTDiscus, CINAHL, the Cochrane Central Register of Controlled Trials (CENTRAL), the Chinese National Knowledge Infrastructure (CNKI), Wanfang, and the Chinese Scientific Journal (VIP) databases was initiated to identify randomized controlled trials (RCTs) and non-randomized comparison studies (NRS) assessing the effect of Tai Chi and Qigong versus various comparison groups on depression and anxiety related outcomes. Study quality was evaluated using a Checklist to Evaluate a Report of a Nonpharmacological Trial (CLEAR-NPT) designed for nonpharmacological trial.

**Results:**

One RCT and six NRS with a total of 772 participants were identified. Some of them were meta-analyzed to examine the pooled effects based on different types of intervention and controls. The results of meta-analyses suggested the effect of Tai Chi was comparable to treatment as usual (TAU) on depression (standardized mean difference (SMD) = − 0.17[− 0.52, 0.17]). Qigong exercise appears to result in improvement on anxiety compared to that of medication (SMD = -1.12[− 1.47, − 0.78]), and no treatment control (SMD = -0.52[− 0.77, − 0.27]).

**Conclusion:**

The findings suggest potentially beneficial effect of Qigong exercise on symptoms of anxiety among individuals with drug abuse. Considering the small number and overall methodological weakness of included studies and lack of RCTs, results should be interpreted with caution and future rigorously designed RCTs are warranted to provide more reliable evidence.

## Background

Substance abuse has become a major public health problem around the world. The World Drug Report 2019 published by the UN Office on Drugs and Crime showed that there had been approximately 35 million people worldwide suffering from substance use disorders, and approximately 585,000 people died from drug abuse in 2017 (UNODC, 2019, p.1) [1]. A direct investigation showed that drug addiction caused 7.4% of the global burden of disease, even more than that caused by AIDS, tuberculosis, diabetes, and other diseases [2]. Drug addiction impairs the cognitive control of drug addicts by damaging the central nervous system, and the drug withdrawal phase involves craving for the drug and substantial lack of self-control [3, 4]. Dependence drugs affect the midbrain dopamine system by stimulating the release as well as inhibiting the uptake of dopamine [5, 6]. The increase of dopamine receptors activates the dopamine activity and function, ultimately resulting in positive reinforced effect [7]. However, once drug addicts reduce the dosage or stop using drugs, the withdrawal syndrome is manifested as a series of physical and mental symptoms [8]. Symptoms of depression and anxiety predominate during the withdrawal phase [9–12], and these challenges the success rate of detoxification and increase the risk of relapse. In severe cases, substance abusers with suicidal ideation may even pay the cost of life [13]. Therefore, it is imperative to improve depression and anxiety in the process of drug addiction treatment and rehabilitation.

Unlike major psychiatric disorders specified in the Diagnostic and Statistical Manual of Mental Disorders Fifth Edition (DSM-5), such as major depression or general anxiety disorder, substance-related symptoms occurring exclusively in the setting of heavy drug intake seem to have different courses, prognoses and treatment approaches [14, 15]. Conventional medications for depression and anxiety should be used cautiously in this setting because they may have potential risk of pharmacological interactions with drug intake [16]. Previous studies have indicated that medication therapy was not ideal for improving depression or anxiety in drug abusers [17–20]. Additionally, the effectiveness of conservative approaches such as cognitive behavioral treatment is not significant and involves relatively high economic and time cost [21, 22]. Physical exercise has been recognized as an important alternative approach to disease prevention and treatment, with its advantages of small side effects, good curative effect and low cost [23]. A meta-analysis conducted by Wang et al. [24] showed that physical exercise can significantly ameliorate anxiety and depressive symptoms in individuals with substance use disorders. There has been accumulating evidence supporting the beneficial effect of physical exercise on various drug withdrawal symptoms, including interpersonal violence, risky sexual behavior, the ability to experience pleasure, mood disorder, and fatigue [25–30].

Beyond conventional forms of physical exercise, the benefits of Tai Chi and Qigong exercise on health promotion have drawn wide attention. Both Tai Chi and Qigong exercise originated from ancient martial arts, which share theoretical roots that are inherent to traditional Chinese medicine [31, 32]. Although there exist some discrepancies in practicing styles and postures, Tai Chi and Qigong exercise are based on the four essential principles of meditative movements proposed by Larkey et al. [33], including some forms of movements or body positioning, explicit attention to the breathing, a meditative state of mind, and a state of deep relaxation. These contributed to the operational definition of Tai Chi and Qigong exercise [34, 35], as adopted in this study. The emphasis on bodily sensations and breathing is contrast to conventional physical exercise with the concentration on actual performance. With regular practice of Tai Chi and Qigong exercise, practitioners may maintain both physical and psychological health.

Previous studies have shown promising effects of Tai Chi or Qigong exercise on improving depression and anxiety among various populations, including healthy adults [36–39], and clinical patients with cancer [40], fibromyalgia [41], and chronic obstructive pulmonary disease [42]. There are some systematic reviews examining the efficacy of mindful-based treatment for alcohol and substance abuse [43, 44]. There is also an increasing number of studies focusing on the effects of Tai Chi and Qigong exercise on drug addicts’ psychological problems, especially anxiety and dependence, during the withdrawal phase, but the literature lacks a systematic review on this subject. Anxiety and depression are especially focused on because they are two of the most common comorbidities in drug abusers [10]. Therefore, the aim of this systematic review was to summarize and synthesize the empirical evidence available from clinical trials on the effectiveness of Tai Chi and Qigong exercise on depression and anxiety in this population.

## Methods

This study complied with the Preferred Reporting Items for Systematic Review and Meta-analyses Statement (PRISMA) [45].

### Search strategy

Electronic searches were conducted in Medline (via PubMed), EMbase (via Ovid), PsychINFO (via Ovid), Eric (via EBSCOhost), SPORTDiscus (via EBSCOhost), CINAHL (via EBSCOhost), the Cochrane Central Register of Controlled Trials (CENTRAL), the Chinese National Knowledge Infrastructure (CNKI), Wanfang, and the Chinese Scientific Journal (VIP) databases from inception through January 2019 to identify all relevant published articles concerning the effect of Tai Chi and Qigong exercise on depression and anxiety of individuals with substance use disorders. *The search words included: Qigong, Qi Gong, Ch’i Kung, Qi-gong, Chi Kung, Chi Chung, Qi Chung, Qi-training, Chi Gong, Qigong Massage, Tai Ji, Tai-ji, Tai Chi, Tai Ji Quan, Taiji, Taijiquan, T’ai Chi, Tai Chi Chuan, Tai Chi Chih, Tai Chi Qigong, Baduanjin, Heroin, Morphine, Cocaine, Methadone, Cannabis, Substance-related Disorders, Opioid, Opiate, Marijuana, Drug Abuse, Drug Usage, Drug Dependence, Drug Addiction, Substance Abuse, Depression, Depressive Disorder, Anxiety, Mental, Psychiatric.* Chinese translations of these terms were used in Chinese databases. A complete record of search strings is provided in the supplementary file. Manually search for reference lists of all included studies and relevant reviews was conducted to further identify relevant studies.

### Eligibility criteria

#### Types of studies

Studies had to be either randomized controlled trials (RCTs) or non-randomized comparison studies (NRS) published in peer-reviewed journals. A study was defined as RCT if the participants were allocated to groups with a clear description of random sequence generation (e.g., using a computer random number generator, coin tossing, drawing of lots); a study was defined as NRS if the allocation of participants was conducted through pseudo-random or non-random sequence generation (e.g., date of birth, date of admission, preference of the participant, or availability of the intervention). Studies that did not involve any comparison group or did not report any comparison results between groups were excluded. Studies were excluded if they were cross-over design, one group pre-post design, cross-sectional or qualitative studies. Additionally, reviews, comments, conference abstracts, and book chapters were excluded.

#### Types of participants

Participants aged 18 years old and over who were diagnosed as illicit drug abusers/dependence based on DSM-3/4/5 were included. Studies simply targeted participants with substance abuse on alcohol or nicotine were excluded. Studies among drug abusers with severe or specific conditions (e.g., cancer, asthma) were excluded.

#### Types of intervention

Studies had to compare the effect of any type of Tai Chi and Qigong exercise with a control group, such as wait list, treatment as usual (TAU) or other types of intervention (e.g., medication). Studies investigating the effect of Tai Chi and Qigong plus another intervention were excluded. Since the focus of this study was on Qigong exercise (internal Qigong), characterized as the coordination of self-directed physical exercise and mediation, studies on external Qigong where participants received healing treatment passively from experienced Qigong masters without physical movements (e.g., Johrei Healing) were excluded.

#### Types of outcome measures

Studies had to measure psychological outcomes with particular emphasis on anxiety and depression using validated instruments. For studies using general psychometric tools to measure various psychological symptoms (e.g., Symptom Checklist 90), only those with outcomes of specific dimensions of depression or anxiety that were provided separately from overall results were included, and only data for depression and/or anxiety dimensions were extracted for meta-analysis. However, those measured general psychological concepts with nonspecific dimensions focusing on depression and anxiety were excluded.

### Study selection and data extraction

Two reviewers independently screened the studies based on the titles, abstracts, and full texts. Discrepancies between the two reviewers (FL, JC) were discussed until consensus was reached. A third reviewer (RL) made the final decision after group discussion if consensus could not be reached. The consistency of abstracts and full-texts screening between the two reviewers was measured using Kappa value proposed by Orwin (2009) [46]. A standardized data extraction form was developed to extract characteristics from each study, including year of publication, location, study design, sample size, age, type of intervention and control group, outcome variables, and main findings. Two reviewers (FL, RL) extracted data of each study independently and disagreement was resolved by discussion.

### Quality assessment

The methodological quality of each included study was assessed by two reviewers independently according to the criteria of a Checklist to Evaluate a Report of a Nonpharmacological Trial (CLEAR-NPT) [54]. CLEAR-NPT was used instead to provide a more comprehensive assessment for those studies where double-blind design was impossible. It assessed the quality based on the following criteria: random sequence generation, allocation concealment, the availability of intervention details, the appropriateness of care providers’ experiences, the adherence of participants, blinding of participants and care providers, blinding of outcome assessors, parallelity of follow-up schedule between groups, and intention-to-treat analysis. Since there are difficulties in executing blinding of participants and care providers in non-pharmacological studies, further assessment criteria serve as alternative evaluation regarding the risk of performance bias if there is no blinding or inadequate blinding. A full description of CLEAR-NPT was provided in the supplementary file. For those studies without providing sufficient information to complete evaluation, the authors were contacted by emails to obtain relevant information. The evaluation “unclear” was provided to specified criteria if no useful response was received from the authors after three rounds of email inquiry.

### Data analysis

Meta-analyses were conducted to explore the effects of interventions upon depression and anxiety. If the study was comparing Tai Chi or Qigong exercise versus two or more controls, the data was kept for each control group separately, and the comparison for that trial was analyzed in the relative control categories. When different instruments were used to measure outcome variables, the effect sizes (ES) in each study was computed using standardized mean differences (SMDs) with 95% confidence interval (CI) between groups. Use of SMD allows for the comparisons across included studies where they used different psychometric instruments to measure the same outcome [55]. The included studies were anticipated to be heterogeneous because of the different characteristics within intervention plan and participants. To account for the potential heterogeneity, a random-effects model was used throughout data synthesis. Random-effects model assumes that included studies are trialed on different populations and each study is calculating a different effect size [56]. I^2^ statistic was used to assess heterogeneity. Studies with an I^2^ statistic of > 75% were considered to have a high degree of heterogeneity; studies with an I^2^ statistic of 50–75% were considered to have a moderate degree of heterogeneity; and studies with an I^2^ statistic of < 50% were considered to have a low degree of heterogeneity. Since less than ten studies were included in each analysis, publication bias was not investigated. All analyses were performed using Comprehensive Meta-analysis Version 2.

## Results

### Study identification

**Fig. 1:**
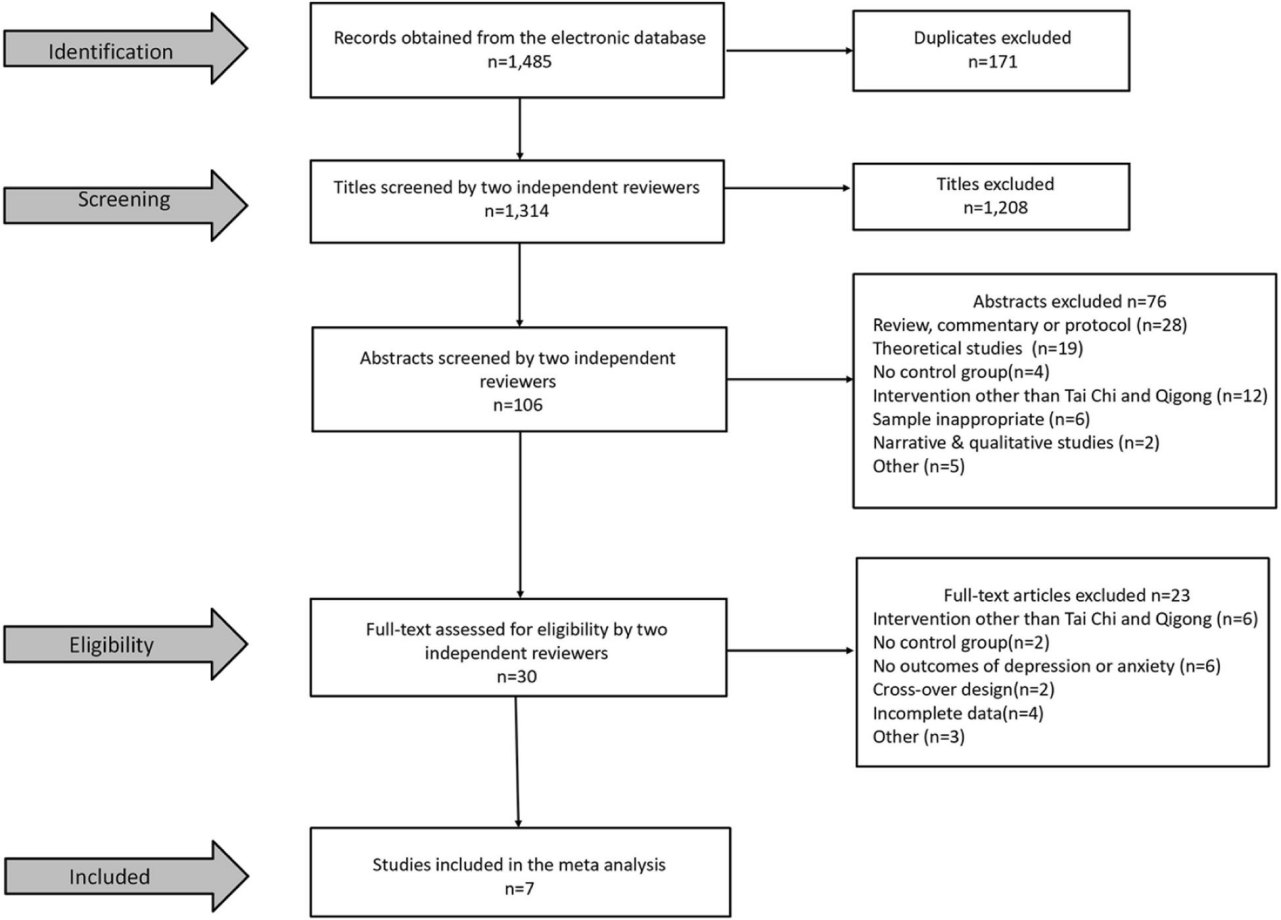
Selection process for included studies

A total of 1485 potentially relevant articles were initially screened in the three Chinese electronic databases and seven English electronic databases based on our literature searching strategy. After removing 171 duplicates and screening based on titles, abstracts, and full-texts, respectively, seven articles were finally included in this review (Fig. 1). Two reviewers were highly consistent in their independent screening process. The kappa coefficient of abstract-based screening reached 0.85 and text-based screening reached 0.84.

### Study characteristics

The characteristics of the included studies are shown in Table 1. Of all included studies, four were published in English peer-reviewed journals, and three in Chinese peer-reviewed journals. Among all included trials, one was performed in USA [48] and six in China [47, 49–53] The participants enrolled in included studies were dependent on heroin, methamphetamine, amphetamine-type stimulant, K powder, ecstasy, synthetic drugs. The sample sizes ranged from 49 to 207, with a total of 772 participants. This included 413 in Tai Chi/Qigong group and 359 in control group. Four studies had 100% female participation [49, 51–53] and one study had 100% male participation [47]. Types of intervention included Qigong exercise (e.g., Baduanjin, Wuqinxi) and Tai Chi (e.g., 24-form); control group included no treatment, TAU, and medication. One trial compared the effect of Qigong versus two types of control, no treatment and medication, respectively [47]. Duration of intervention ranged from 10 days to 24 weeks. All studies examined the effect of Tai Chi and Qigong exercise immediately after intervention, but few studies provided follow-up measurement of outcomes. Five studies measured anxiety variables [47, 48, 50–52] and five studies measured depression variables [48, 49, 51–53]. The anxiety scales used included Spielberger State-Trait Anxiety Inventory-State, Hamilton Anxiety Scale, Self-rating Anxiety Scale, and Symptom Checklist 90; the depression scales used included Hamilton Rating Scale for Depression, Center for Epidemiological Studies Depression Scale, Self-rating Depression Scale, and Symptom Checklist 90. Among them, symptom Checklist 90 is a self-report symptom inventory to measure nine primary symptoms dimensions including depression and anxiety. Only these two dimensions of interest were included for analyses.

**Table 1:** Characteristics of included studies of the effects of Tai Chi and Qigong exercise on symptoms of depression and anxiety

Study	Study design; location	Study participants	Sample size (Mean age ± SD)	Intervention	Control	Duration	Outcome measures	Results
Li et al., 2002 [47]	NRS, Jiangsu, China	Males with dependence on heroin	Exp: 34 (33.3 ± 6.5) Con 1: 26 (31.9 ± 5.9) Con 2: 26 (31.7 ± 6.1)	Qigong (2–2.5 h, once per day)	(1) Medication, (2) No treatment	1wk + 3d	HAS	Exp vs. Con 1: *p* < 0.001 Exp vs. Con 2: *p* < 0.01
Chen et al., 2010 [48]	NRS, USA	Individuals with drug abuse	Exp: 126 (35.9 ± 10.9) Con: 81 (30.7 ± 8.9)	Qigong (1 h, twice per day, five or more days per week)	TAU	2wk	(1) CES (2) STAI	(1) *p* > 0.05 (2) *p* > 0.05
Li et al.,2013 [49]	NRS, Yunnan, China	Females with drug dependence	Exp: 36 (30.7 ± 6.3) Con: 34 (30.7 ± 6.3)	Tai Chi (1 h, once every two days)	TAU	24wk	HRSD	*p* = 0.243
Huang et al., 2015 [50]	NRS, Zhuhai, China	Individuals with dependence on heroin	Exp: 50 (35.26 ± 12.22) Con: 50 (35.21 ± 12.12)	Qigong (Baduanjin, 30 min, twice a day)	Medication	20wk	SAS	*p*<0.05
Geng et al., 2016 [51]	NRS, Shanghai, China	Females with drug dependence	Exp: 30 (34 ± 7) Con: 30 (38 ± 5)	Tai Chi (24-form, 45 min, five sessions per week)	TAU	12wk	SCL-90	Depression: *p* > 0.05 Anxiety: *p* > 0.05
Fu et al., 2016 [52]	NRS, Anhui, China	Females with drug dependence	Exp: 100 (28.3 ± 7.83) Con: 100 (27.99 ± 8.17)	Qigong (Wuqinxi, 30 min, once per day)	No treatment	20wk	(1) SAS (2) SDS	(1) *p* = 0.000 (2) *p* = 0.003
Zhu et al., 2018 [53]	RCT, Shanghai, China	Females with dependence on amphetamine-type stimulant	Exp: 37 (?? ±??) Con: 12 (?? ±??)	Tai Chi (24-form, 1 h, five sessions per week for the first 3 months, and three times per week for next 3 months)	TAU	24wk	SDS	*p*>0.05

*RCT* Randomized controlled trial, *NRS* Non-randomized comparison study, *Exp* experiment group, *Con* Control group, *TAU* Treatment as usual;??: not provided in the text; *HAS* Hamilton Anxiety Scale, *HRSD* Hamilton Rating Scale for Depression, *CES* CES Depression Scale, *STAI* Spielberger State-Trait Anxiety Inventory-State only, *SDS* Self-Rating Depression Scale, *SAS* Self—Rating Anxiety Scale, *SCL-90* Symptom Checklist 90

### Quality assessment

We evaluated each study based on ten criteria (Table 2). One study complied with the adequate procedure of randomized allocation using computer-generated random numbers [53]. The remaining six studies allocated the participants through pseudo-random sequence generation [47, 48, 52] or did not clarify the method of random sequence generation [49–51]. The concealment of allocation and care providers’ experience were not explicitly provided in all of the included studies. The details of the intervention administered to each group were available in all studies. Although none of the studies blinded participants and care providers adequately, three of them provided the same treatments and care in each group and the withdrawals in each group showed no significant difference [47, 49, 50]. The blinding of outcome assessors was adequate in three studies [47, 49, 53]. ‘N/A (not applicable)’ was marked for the remaining four studies because the outcome measures (i.e., anxiety and depression) were self-reported by the participants, which were less likely to be influenced by lacking of assessor blinding. Six studies executed the follow-up schedule exactly the same in each group [48–53]. The main outcomes were analyzed according to intention-to-treat principle in only two trials [47, 52].

**Table 2:** Critical appraisal of included studies

Criteria	Study reference						
	Li 2002 [47]	Chen 2010 [48]	Li 2013 [49]	Huang 2015 [50]	Geng 2016 [51]	Fu 2016 [52]	Zhu 2018 [53]
1. Was the generation of allocation adequate?	N	N	U	U	U	N	Y
2. Was the treatment allocation concealed?	N	N	U	U	U	N	N
3. Were details of the intervention administered to each group made available?	Y	Y	Y	Y	Y	Y	Y
4. Were care providers’ experience or skills in each arm appropriate?	U	N	U	U	U	U	U
5. Was participant (i.e., patients) adherence assessed quantitatively?	U	Y	U	U	N	U	U
6. Were participants adequately blinded? if no, go to point 6.1 and 6.2	N	N	N	N	N	N	N
6.1 Were other treatments and care (i.e. co-interventions) the same in each randomized group?	Y	N	Y	Y	U	U	Y
6.2 Were withdrawals and lost-to-follow-up the same in each randomized group?	Y	N	Y	Y	Y	Y	N
7. Were care providers for the participants adequately blinded? if no, go to point 7.1 and 7.2	N	N	N	N	N	N	N
7.1 Were other treatments and care (i.e. co-interventions) the same in each randomized group?	Y	N	Y	Y	U	U	Y
7.2 Were withdrawals and lost-to-follow-up the same in each randomized group?	Y	N	Y	Y	Y	Y	N
8. Were outcome assessors adequately blinded to assess the primary outcomes? If no, go to 8.1	Y	N/A	Y	N/A	N/A	N/A	Y
8.1 If outcome assessors were not adequately blinded, were specific methods used to avoid ascertainment bias?	N/A	N/A	N/A	N/A	N/A	N/A	N/A
9. Was the follow-up schedule the same in each group? (parallel design)	U	Y	Y	Y	Y	Y	Y
10. Were the main outcomes analyzed according to the intention-to-treat principle?	Y	N	N	N	N	Y	N

*Y* Yes, *N* No, *N/A* Not appropriate, *U* Unable to determine

### Effects of tai chi and Qigong exercise on depressive symptoms

**Fig. 2:**
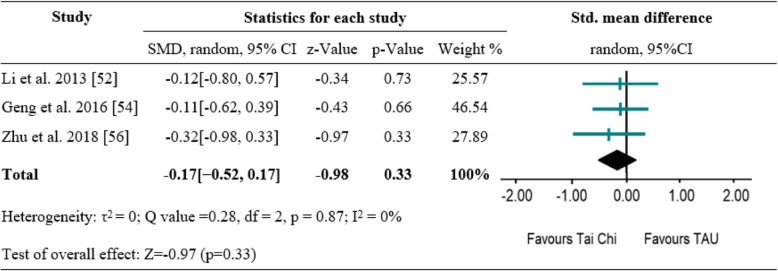
A meta-analysis of comparing Tai Chi to treatment as usual for changes in depressive symptoms

Of the included seven studies, five measured depressive symptoms as primary outcome [48, 49, 51–53]. Three studies compared the effect of Tai Chi versus TAU on depressive symptoms [49, 51, 53]; two studies compared the effect of Qigong versus TAU [48] and no treatment control [52] on depression. Because of heterogeneity of types of intervention and control across the included studies, synthesis of the results of these studies directly would be inappropriate. Therefore, subsequent meta-analysis was conducted using the three studies [49, 51, 53] with the same type of intervention and control to examine the pooled effect. The random effects analysis was conducted to merge the results due to discrepancies of outcome measures. All trials suggested no between-group difference. The pooled results showed no significant difference between Tai Chi and TAU on depressive symptoms (SMD = -0.17[− 0.52, 0.17], *p* = 0.33) with high degree of homogeneity (I^2^ = 0%) (Fig. 2). Similarly, one study showed Qigong exercise did not differ significantly from TAU on depressive symptoms (SMD = -0.11[− 0.39, 0.17], *p* = 0.42) [53]. However, Qigong exercise resulted in significant improvement in depressive symptoms compared with no treatment control (SMD = -0.47[− 0.75, − 0.19], *p* < 0.001) [52].

### Effects of tai chi and Qigong exercise on anxiety symptoms

**Fig. 3:**
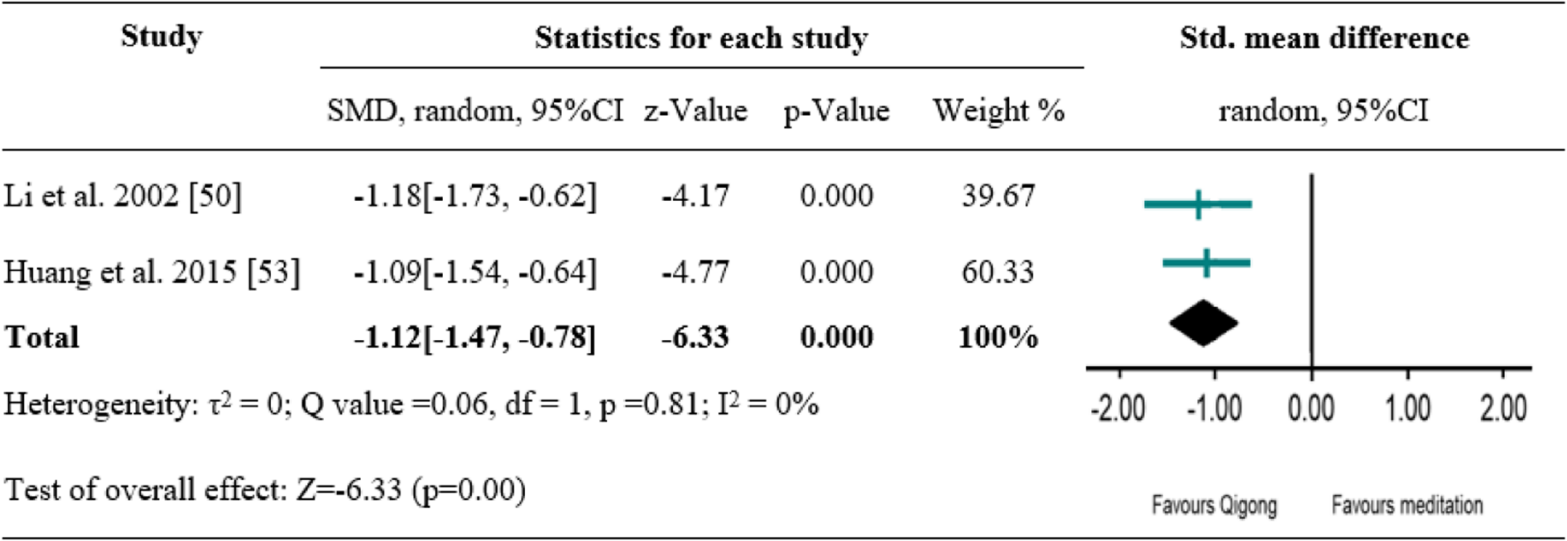
A meta-analysis of comparing Qigong to medication control for changes in anxiety symptoms

**Fig. 4:**
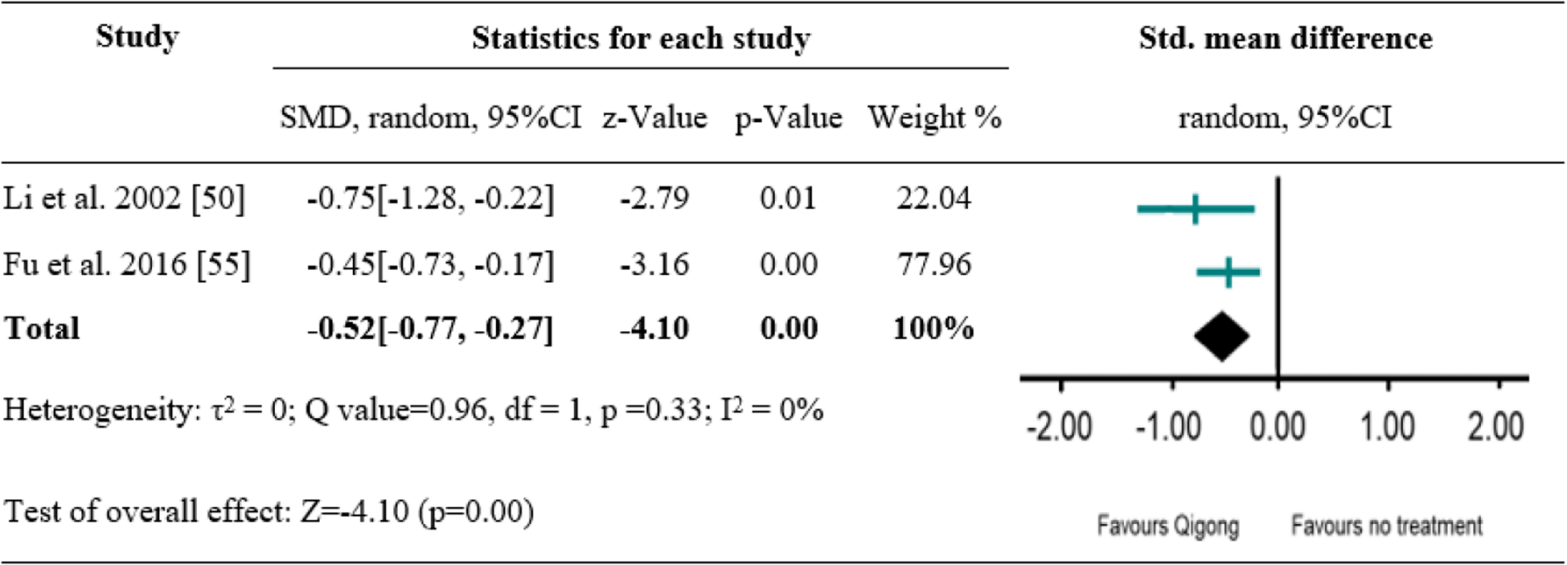
A meta-analysis of comparing Qigong to no treatment for changes in anxiety symptoms

Of the included seven studies, five measured anxiety symptoms as primary outcome [47, 48, 50–52]. Among them, two studies evaluated the effect of Qigong versus medication on anxiety symptoms [47, 50], two another studies evaluated the effect of Qigong versus no treatment control [47, 52]; the remaining two studies compared the effect of Qigong and Tai Chi, respectively, with that of TAU [48, 51]. Subsequent meta-analysis was conducted to examine the pooled effect of Qigong versus medication as well as Qigong versus no treatment on anxiety outcomes. The random effects analysis was conducted to merge the results due to discrepancies of outcome measures. A significantly favourable effect of Qigong versus medication was found (SMD = -1.12[− 1.47, − 0.78], *p* < 0.001), with high degree of homogeneity (I^2^ = 0%) (Fig. 3). A significant difference between Qigong and no treatment control was also shown (SMD = -0.52[− 0.77, − 0.27], *p* < 0.001), with high degree of homogeneity (I^2^ = 0%) (Fig. 4). Compared to TAU, Qigong and Tai Chi exercise did not result in significant improvements in anxiety symptoms: Qigong (SMD = -0.10[− 0.38, 0.18], *p* = 0.50) [48], Tai Chi (SMD = -0.32[− 0.82, 0.19], *p* = 0.23) [51].

## Discussion

To the best of our knowledge, this is the first systematic review focusing on the effect of Tai Chi and Qigong exercise intervention on symptoms of depression and anxiety of drug addicts. The current review conducted a systematic literature search and included relevant studies based on standardized inclusion and exclusion criteria. The novel findings based on available evidence demonstrate that both Tai Chi and Qigong exercise may not differ significantly from that of TAU on reducing depression and anxiety. However, a favourable effect of Qigong exercise on reducing anxiety was found compared to medication and no treatment group. Existing evidence might also suggest a promising effect of Qigong exercise on depression when compared to no treatment control. More studies with high homogeneity in methodology are needed to confirm these findings. Previous systematic review and meta-analysis found physical exercise could be an effective treatment on depression and anxiety symptoms for substance use disorders [24]. Importantly, our study specifically focuses on Tai Chi and Qigong exercise and results were reported based on different intervention and comparison group, which is different from previous studies that assessed the effectiveness of general physical exercise on psychological symptoms and did not analyze different types of exercise (e.g., walking, cycling, Qigong, yoga) separately as subgroups [24, 57, 58].

Considering that antidepressant medications for addicts with drug abstinence are not recommended in clinical settings to avoid unnecessary drug side effects, and that there is little convincing evidence to support the effectiveness of standard antidepressant medications in substance-related mood changes, meditative movements like Tai Chi and Qigong exercise could probably be a promising alternative to conventional treatment in terms of improving the affective state of drug addicts. Furthermore, Qigong’s slow gentle meditative movements are of low intensity and may be preferred among drug addicts who are in a less healthy condition. Notably, this review did not reveal the superiority of Tai Chi and Qigong exercise over TAU on depression and anxiety symptoms. Feasible explanation could be that drug abusers in TAU group received standard care, which usually involves a certain amount of physical exercise (e.g., relaxation training, broadcast calisthenics, rehabilitation exercise) or even similar exercise intensity and frequency as those of a Qigong group [47, 50]. It is possible that the inclusion of physical exercise routine in TAU group may play a similar role as an intervention. Compared to no treatment control, the significant effect of Qigong exercise on depression may partially account for this explanation. Future studies comparing the effect of Tai Chi or Qigong exercise versus conventional physical exercise on depression and anxiety are encouraged. Additionally, it is interesting to find that Qigong exercise could be effective in improving depression and anxiety when compared to medication and no treatment control. A previous meta-analysis stated that Qigong appears to be effective in improving depressive symptoms but not Tai Chi [59], which could probably because that Qigong emphasizes more on meditative state of the body than Tai Chi, and this may partially explain their differences in therapeutic effect [59]. However, it is premature to come to this conclusion at this point because no evidence was provided regarding the effects of Tai Chi versus medication or no treatment control on symptoms of depression and anxiety.

Possible mechanisms of Tai Chi and Qigong exercise on depression and anxiety among individuals with substance abuse disorder can be explained from the following perspectives. First, Tai Chi and Qigong exercise can decrease physiological arousal and promote relaxation, which, in turn, mitigates the anxiety sensitivity and the possibility of relapse [60]. Second, mindful concentration emphasized by Tai Chi and Qigong exercise strengthens the brain circuits involved in the regulation of prefrontal cortex. The repeated activation of prefrontal cortex, that occupies neurocognitive resources, may mediate the “pleasure dissonance” and facilitate the automation of cognitive control of drug addicts [60]. Third, reconstruction of reward system theory claimed that the perception and sensation of happiness experience brought from practicing Tai Chi and Qigong, as an alternative where awards were gained spontaneously, may reconstruct the source of rewarding and thereby resolve the “pleasure dissonance” [61]. Additionally, there has been magnetic resonance imaging evidence showing that the mindfulness training can create new connections of brain nerve cells of prefrontal lobe, and decrease activities of the limbic system responsible for emotion response regulation [62, 63]. Last, the practice of Tai Chi and Qigong in groups may promote individuals’ social interaction with their partners, which can facilitate the perception of positive expectation and social support from each other, and ultimately benefit their mental health [36].

The present review has several potential limitations. First, the limited number and overall low quality of the included studies sets up a barrier for conducting a rigorous meta-analysis. Therefore, the potential beneficial effect of Qigong exercise is not conclusive and the findings should be viewed cautiously. Second, the notable varieties of study methodology contributing to heterogeneity across studies, such as various dose of intervention (e.g., length, frequency, and duration of practice), outcome measurements, type of drug addiction, and gender difference, could not be assessed. Nevertheless, this heterogeneity may help increase the external validity of the findings. Another limitation is that this review did not include unpublished or ongoing research and could not assess the long-term effect as the included studies did not provide follow-up measurements of depression and anxiety outcomes. Furthermore, it is difficult to address the most optimized mode of Tai Chi and Qigong intervention, because limited number of studies prevented from comparing different style, intensity, frequency and duration of intervention. Future studies with larger sample size, rigorous RCT design, various type of Tai Chi and Qigong exercise compared with other intervention modalities (e.g., psychological education, aerobic exercise) are necessary to fill in these gaps. To design a more rigorous RCT, allocation of participants into groups based on random sequence generation and with proper allocation concealment should be taken into account to minimize experimenter bias. As drug addicts usually receive scheduled treatment at drug rehabilitation center, future studies should consider the duration of treatment when designing the intervention program to avoid losing participants halfway through the trial. Regardless of these limitations, this systematic review takes advantage of tangible information from studies to account for the insufficiency of included trials with high heterogeneity that may impact the findings.

## Conclusions

This systematic review, based on existing literature of both randomized and non-randomized controlled trials, shows that Tai Chi and Qigong exercise appear to have similar beneficial effect as usual care involving conventional exercise on the improvement of depression and anxiety in individuals with substance abuse disorders. Compared with no treatment control or medication therapy, Qigong exercise seems to show potential advantages in reducing depression and anxiety, suggesting the possibility of Qigong as an alternative medical therapy to improve substance-related affective disorders. However, these results are not conclusive due to the overall poor quality and limited number of the reviewed studies.

## Supplementary information

**Additional file 1.** Search terms used in each database. 2 Checklist to Evaluate a Report of a Nonpharmacological Trial.

## Data Availability

All data generated during this study are included in this published article.

## Abbreviations

CNKI: Chinese National Knowledge Infrastructure
RCT: Randomized controlled trials
NRS: Non-randomized comparison studies
CLEAR-NPT: A Checklist to Evaluate a Report of a Nonpharmacological Trial
TAU: Treatment as usual
DSM-5: The Diagnostic and Statistical Manual of Mental Disorders Fifth Edition
PRISMA: Preferred Reporting Items for Systematic Review and Meta-analyses Statement
ES: Effect sizes
SMDs: Standardized mean differences
CI: Confidence interval
