# Supplementary material for: The effect of tai chi and Qigong exercise on depression and anxiety of individuals with substance use disorders: a systematic review and meta-analysis
Source: BMC Complement Med Ther. 2020 May 29;20:161. doi: 10.1186/s12906-020-02967-8 (PMC7260819; doi:10.1186/s12906-020-02967-8)
Supplement: Supplementary file 1 — Additional file 1. Search terms used in each database. 2 Checklist to Evaluate a Report of a Nonpharmacological Trial. [file 12906_2020_2967_MOESM1_ESM.docx]

**Supplementary materials**

**List of content:**

–Additional file 1. Search terms used in each database

–Additional file 2. Checklist to Evaluate a Report of a Nonpharmacological Trial (CLEAR-NPT)

Additional file 1.

Table 1. Search strategy in Medline via Pubmed.

| Search | Query | Items found |
| --- | --- | --- |
| #1 | Search ((((((((((((((((((((((Qigong[MeSH Terms]) OR Tai Ji[MeSH Terms]) OR Qigong[Title/Abstract]) OR Tai Ji[Title/Abstract]) OR Ch'i Kung[Title/Abstract]) OR Qi-gong[Title/Abstract]) OR Chi Kung[Title/Abstract]) OR Chi Chung[Title/Abstract]) OR Qi Chung[Title/Abstract]) OR Qi-training[Title/Abstract]) OR Chi Gong[Title/Abstract]) OR Qigong Massage[Title/Abstract]) OR Tai-ji[Title/Abstract]) OR Tai Chi[Title/Abstract]) OR Tai Ji Quan[Title/Abstract]) OR Taiji[Title/Abstract]) OR Taijiquan[Title/Abstract]) OR T'ai Chi[Title/Abstract]) OR Tai Chi Chuan[Title/Abstract]) OR Tai Chi Chih[Title/Abstract]) OR Tai Chi Qigong[Title/Abstract]) OR Baduanjin[Title/Abstract]) OR Qi Gong[Title/Abstract] | 2564 |
| #2 | Search (((((((((((((((((((Heroin[MeSH Terms]) OR Morphine[MeSH Terms]) OR Cocaine[MeSH Terms]) OR Methadone[MeSH Terms]) OR Cannabis[MeSH Terms]) OR Substance-Related Disorders[MeSH Terms]) OR Opioid[Title/Abstract]) OR Opiate[Title/Abstract]) OR Marijuana[Title/Abstract]) OR Drug Abuse[Title/Abstract]) OR Drug Usage[Title/Abstract]) OR Drug Dependence[Title/Abstract]) OR Drug Addiction[Title/Abstract]) OR Substance Abuse[Title/Abstract]) OR Heroin[Title/Abstract]) OR Morphine[Title/Abstract]) OR Cocaine[Title/Abstract]) OR Methadone[Title/Abstract]) OR Cannabis[Title/Abstract]) OR Substance-Related Disorders[Title/Abstract] | 433589 |
| #3 | Search ((((((((((Depression[MeSH Terms]) OR Depressive Disorder[MeSH Terms]) OR Anxiety[MeSH Terms]) OR Depression[All Fields]) OR Depressive Disorder[All Fields]) OR Anxiety[All Fields]) OR Depress*[Title/Abstract]) OR Anxi*[Title/Abstract]) OR Mental[All Fields]) OR Psychiatric[All Fields]) OR Psych*[Title/Abstract] | 1595808 |
| #4 | #1 and #2 and #3 | 20 |

Table 2. Search strategy in Embase and PsychInfo via Ovid.

| Search | Query | Items found |
| --- | --- | --- |
| 1 | exp Qigong/ | 699 |
| 2 | exp Tai Ji/ | 2817 |
| 3 | Qigong.ab,kw,ti. | 1262 |
| 4 | Tai Ji.ab,kw,ti. | 99 |
| 5 | Qi Gong.ab,kw,ti. | 243 |
| 6 | Ch'i Kung.ab,kw,ti. | 26 |
| 7 | Qi-gong.ab,kw,ti. | 243 |
| 8 | Chi Kung.ab,kw,ti. | 26 |
| 9 | Chi Chung.ab,kw,ti. | 9 |
| 10 | Qi Chung.ab,kw,ti. | 0 |
| 11 | Qi-training.ab,kw,ti. | 167 |
| 12 | Chi Gong.ab,kw,ti. | 13 |
| 13 | Qigong Massage.ab,kw,ti. | 19 |
| 14 | Tai Chi.ab,kw,ti. | 2936 |
| 15 | Tai Ji Quan.ab,kw,ti. | 48 |
| 16 | Taiji.ab,kw,ti. | 133 |
| 17 | Taijiquan.ab,kw,ti. | 66 |
| 18 | T'ai Chi.ab,kw,ti. | 2936 |
| 19 | Tai Chi Chuan.ab,kw,ti. | 304 |
| 20 | Tai Chi Chih.ab,kw,ti. | 45 |
| 21 | Tai Chi Qigong.ab,kw,ti. | 87 |
| 22 | Baduanjin.ab,kw,ti. | 101 |
| 23 | Tai-ji.ab,kw,ti. | 99 |
| 24 | 1 or 2 or 3 or 4 or 5 or 6 or 7 or 8 or 9 or 10 or 11 or 12 or 13 or 14 or 15 or 16 or 17 or 18 or 19 or 20 or 21 or 22 or 23 | 5223 |
| 25 | exp Heroin/ | 26078 |
| 26 | exp Morphine/ | 106740 |
| 27 | exp Cocaine/ | 67593 |
| 28 | exp Methadone/ | 33017 |
| 29 | exp Cannabis/ | 41442 |
| 30 | exp Substance-Related Disorders/ | 227305 |
| 31 | Heroin.ab,kw,ti. | 27767 |
| 32 | Morphine.ab,kw,ti. | 74537 |
| 33 | Cocaine.ab,kw,ti. | 67283 |
| 34 | Methadone.ab,kw,ti. | 25399 |
| 35 | Cannabis.ab,kw,ti. | 32966 |
| 36 | Substance-Related Disorders.ab,kw,ti. | 2140 |
| 37 | Opioid.ab,kw,ti. | 120630 |
| 38 | Opiate.ab,kw,ti. | 32832 |
| 39 | Marijuana.ab,kw,ti. | 26674 |
| 40 | Drug Abuse.ab,kw,ti. | 37294 |
| 41 | Drug Usage.ab,kw,ti. | 3099 |
| 42 | Drug Dependence.ab,kw,ti. | 8499 |
| 43 | Drug Addiction.ab,kw,ti. | 15143 |
| 44 | Substance Abuse.ab,kw,ti. | 65746 |
| 45 | 25 or 26 or 27 or 28 or 29 or 30 or 31 or 32 or 33 or 34 or 35 or 36 or 37 or 38 or 39 or 40 or 41 or 42 or 43 or 44 | 617720 |
| 46 | exp Depression/ | 483081 |
| 47 | exp Depressive Disorder/ | 457789 |
| 48 | exp Anxiety/ | 269322 |
| 49 | Depression.ab,kw,ti. | 691300 |
| 50 | Depressive Disorder.ab,kw,ti. | 63432 |
| 51 | Anxiety.ab,kw,ti. | 451341 |
| 52 | "Depress*".ab,kw,ti. | 894807 |
| 53 | "Anxi*".ab,kw,ti. | 491988 |
| 54 | Mental.ab,kw,ti. | 802141 |
| 55 | Psychiatric.ab,kw,ti. | 439315 |
| 56 | "Psych*".ab,kw,ti. | 2210864 |
| 57 | 46 or 47 or 48 or 49 or 50 or 51 or 52 or 53 or 54 or 55 or 56 | 3476392 |
| 58 | 24 and 45 and 57 | 52 |

Table 3. Search strategy in CINAHL, ERIC, and SPORTDiscus via EBSCOhost.

| **#** | **Query** | **Results** |
| --- | --- | --- |
| S1 | (MH “Qigong”) | 382 |
| S2 | (MH “Tai Chi”) | 1,236 |
| S3 | TI “Qigong” OR AB “Qigong” OR TI “Tai Ji” OR AB “Tai Ji” OR TI “Tai Chi” OR AB “Tai Chi” OR TI “Ch'i Kung” OR AB “Ch'i Kung” OR TI “Qi-gong” OR AB “Qi-gong” OR TI “Chi Kung” OR AB “Chi Kung” | 3,427 |
| S4 | TI “Qi Gong” OR AB “Qi Gong” OR TI “Chi Chung” OR AB “Chi Chung” OR TI “Qi Chung” OR AB “Qi Chung” OR TI “Qi-training” OR AB “Qi-training” OR TI “Chi Gong” OR AB “Chi Gong” OR TI “Qigong Massage” OR AB “Qigong Massage” | 246 |
| S5 | TI “Tai-ji” OR AB “Tai-ji” OR TI “Tai Ji Quan” OR AB “Tai Ji Quan” OR TI “Taiji” OR AB “Taiji” OR TI “Taijiquan” OR AB “Taijiquan” OR TI “T'ai Chi” OR AB “T'ai Chi” OR TI “Tai Chi Chuan” OR AB “Tai Chi Chuan” | 1,104 |
| S6 | TI “Tai Chi Chih” OR AB “Tai Chi Chih” OR TI “Tai Chi Qigong” OR AB “Tai Chi Qigong” OR TI “Baduanjin” OR AB “Baduanjin” | 228 |
| S7 | S1 OR S2 OR S3 OR S4 OR S5 OR S6 | 4,478 |
| S8 | (MH “Heroin”) | 1,538 |
| S9 | (MH “Morphine”) | 3,343 |
| S10 | (MH “Cocaine”) | 2,089 |
| S11 | (MH “Methadone”) | 2,795 |
| S12 | (MH “Cannabis”) | 4,913 |
| S13 | TI “Heroin” OR AB “Heroin” OR TI “Morphine” OR AB “Morphine” OR TI “Cocaine” OR AB “Cocaine” OR TI “Methadone” OR AB “Methadone” OR TI “Cannabis” OR AB “Cannabis” OR TI “Substance-Related Disorders” OR AB “Substance-Related Disorders” | 37,631 |
| S14 | TI “Opioid” OR AB “Opioid” OR TI “Opiate” OR AB “Opiate” OR TI “Marijuana” OR AB “Marijuana” OR TI “Drug Abuse” OR AB “Drug Abuse” OR TI “Drug Usage” OR AB “Drug Usage” OR TI “Drug Dependence” OR AB “Drug Dependence” | 59,047 |
| S15 | TI “Drug Addiction” OR AB “Drug Addiction” OR TI “Substance Abuse” OR AB “Substance Abuse” | 23,564 |
| S16 | S8 OR S9 OR S10 OR S11 OR S12 OR S13 OR S14 OR S15 | 103,089 |
| S17 | (MH “Depression”) | 58,732 |
| S18 | (MH “Anxiety”) | 17,622 |
| S19 | TI “Depression” OR AB “Depression” OR TI “Depressive Disorder” OR AB “Depressive Disorder” OR TI “Anxiety” OR AB “Anxiety” OR TI “Depress*” OR AB “Depress*” OR TI “Anxi*” OR AB “Anxi*” OR TI “Mental” OR AB “Mental” | 400,354 |
| S20 | TI “Psychiatric” OR AB “Psychiatric” OR TI “Psych*” OR AB “Psych*” | 429,959 |
| S21 | S17 OR S18 OR S19 OR S20 | 731,259 |
| S22 | S7 AND S16 AND S21 | 14 |

Table 4. Search strategy in the Cochrane Central Register of Controlled Trials (CENTRAL)

| Search | Query | Items found |
| --- | --- | --- |
| #1 | MeSH descriptor: [Qigong] explode all trees | 59 |
| #2 | (Qigong):ti,ab,kw (Word variations have been searched) | 433 |
| #3 | (Ch'i Kung):ti,ab,kw (Word variations have been searched) | 0 |
| #4 | (Qi-gong):ti,ab,kw (Word variations have been searched) | 50 |
| #5 | (Chi Kung):ti,ab,kw (Word variations have been searched) | 11 |
| #6 | (Qi Gong):ti,ab,kw (Word variations have been searched) | 54 |
| #7 | (Chi Chung):ti,ab,kw (Word variations have been searched) | 5 |
| #8 | (Qi Chung):ti,ab,kw (Word variations have been searched) | 1 |
| #9 | (Qi-training):ti,ab,kw (Word variations have been searched) | 11 |
| #10 | (Chi Gong):ti,ab,kw (Word variations have been searched) | 17 |
| #11 | (Qigong Massage):ti,ab,kw (Word variations have been searched) | 13 |
| #12 | MeSH descriptor: [Tai Ji] explode all trees | 332 |
| #13 | (Tai Ji):ti,ab,kw (Word variations have been searched) | 354 |
| #14 | (Tai-ji):ti,ab,kw (Word variations have been searched) | 349 |
| #15 | (Tai Chi):ti,ab,kw (Word variations have been searched) | 1097 |
| #16 | (Tai Ji Quan):ti,ab,kw (Word variations have been searched) | 24 |
| #17 | (Taiji):ti,ab,kw (Word variations have been searched) | 36 |
| #18 | (Taijiquan):ti,ab,kw (Word variations have been searched) | 83 |
| #19 | (T'ai Chi):ti,ab,kw (Word variations have been searched) | 37 |
| #20 | (Tai Chi Chuan):ti,ab,kw (Word variations have been searched) | 106 |
| #21 | (Tai Chi Chih):ti,ab,kw (Word variations have been searched) | 33 |
| #22 | (Tai Chi Qigong):ti,ab,kw (Word variations have been searched) | 71 |
| #23 | (Baduanjin):ti,ab,kw (Word variations have been searched) | 98 |
| #24 | #1 OR #2 OR #3 OR #4 OR #5 OR #6 OR #7 OR #8 OR #9 OR #10 OR #11 OR #12 OR #13 OR #14 OR #15 OR #16 OR #17 OR #18 OR #19 OR #20 OR #21 OR #22 OR #23 | 1672 |
| #25 | MeSH descriptor: [Heroin] explode all trees | 299 |
| #26 | (Heroin):ti,ab,kw (Word variations have been searched) | 1707 |
| #27 | MeSH descriptor: [Morphine] explode all trees | 4749 |
| #28 | (Morphine):ti,ab,kw (Word variations have been searched) | 13469 |
| #29 | MeSH descriptor: [Cocaine] explode all trees | 909 |
| #30 | (Cocaine):ti,ab,kw (Word variations have been searched) | 3311 |
| #31 | MeSH descriptor: [Methadone] explode all trees | 1189 |
| #32 | (Methadone):ti,ab,kw (Word variations have been searched) | 2812 |
| #33 | MeSH descriptor: [Cannabis] explode all trees | 297 |
| #34 | (Cannabis):ti,ab,kw (Word variations have been searched) | 2289 |
| #35 | MeSH descriptor: [Substance-Related Disorders] explode all trees | 14094 |
| #36 | (Substance-Related Disorders):ti,ab,kw (Word variations have been searched) | 3712 |
| #37 | (Opioid):ti,ab,kw (Word variations have been searched) | 21803 |
| #38 | (Opiate):ti,ab,kw (Word variations have been searched) | 6249 |
| #39 | (Marijuana):ti,ab,kw (Word variations have been searched) | 1842 |
| #40 | (Drug Abuse):ti,ab,kw (Word variations have been searched) | 7643 |
| #41 | (Drug Usage):ti,ab,kw (Word variations have been searched) | 4090 |
| #42 | (Drug Dependence):ti,ab,kw (Word variations have been searched) | 48232 |
| #43 | (Drug Addiction):ti,ab,kw (Word variations have been searched) | 5457 |
| #44 | (Substance Abuse):ti,ab,kw (Word variations have been searched) | 5969 |
| #45 | #25 OR #26 OR #27 OR #28 OR #29 OR #30 OR #31 OR #32 OR #33 OR #34 OR #35 OR #36 OR #37 OR #38 OR #39 OR #40 OR #41 OR #42 OR #43 OR #44 | 97671 |
| #46 | MeSH descriptor: [Depression] explode all trees | 10762 |
| #47 | (Depression):ti,ab,kw (Word variations have been searched) | 79844 |
| #48 | MeSH descriptor: [Depressive Disorder] explode all trees | 11061 |
| #49 | (Depressive Disorder):ti,ab,kw (Word variations have been searched) | 34234 |
| #50 | MeSH descriptor: [Anxiety] explode all trees | 7510 |
| #51 | (Anxiety):ti,ab,kw (Word variations have been searched) | 46562 |
| #52 | (Mental):ti,ab,kw (Word variations have been searched) | 54320 |
| #53 | (Psychiatric):ti,ab,kw (Word variations have been searched) | 24521 |
| #54 | #46 OR #47 OR #48 OR #49 OR #50 OR #51 OR #52 OR #53 | 147394 |
| #55 | #24 AND #45 AND # 54 | 2 |

Additional file 2. Checklist to Evaluate a Report of a Nonpharmacological Trial (CLEAR-NPT)

|  | **Criterion** | **Yes** | **No** | **Unclear** | **Comment/Note** |
| --- | --- | --- | --- | --- | --- |
| 1 | Was the generation of allocation sequences adequate? |  |  |  | The investigator should describe a random component in sequence generation process. |
| 2 | Was the treatment allocation concealed? |  |  |  | Adequate concealment of allocations prior to assignment |
| 3 | Were details of the intervention administered to each group stated or made available? |  |  |  | Did the investigator describe the intervention administered to each group clearly? |
| 4 | Were care providers experiences or skills in each arm (group) appropriate? |  |  |  | Did the investigator describe the experiences or skills of care providers in each group? |
| 5 | Was participant adherence or compliance assessed quantitatively? |  |  |  | Did the investigator quantitatively measured how the participants followed the intervention protocol? |
| 6 | Were participants adequately blinded?   - If NO, please go to 7.1. and 7.2. |  |  |  | The answer is usually NO in nonpharmacological studies |
| 6.1 | Were other treatments and care (i.e., co-interventions) the same in each randomized group? |  |  |  | If there exited co-interventions, did each group get same amount of time/attention of co-interventions? |
| 6.2 | Were withdrawals and lost-to-follow-up the same in each randomized group? |  |  |  | Calculate the difference of drop-out to see if it is statistically significant. |
| 7 | Were care providers for the participants adequately blinded?   - If NO, please go to 8.1. and 8.2. |  |  |  | The answer is usually NO in nonpharmacological studies |
| 7.1 | Were all other treatments and care (co-interventions) the same in each randomized group? |  |  |  | Were the care providers of each group had equivalent experiences of training? |
| 7.2 | Were withdrawals and lost-to-follow up the same in each randomized group? |  |  |  | Calculate the difference of drop-out to see if it is statistically significant. |
| 8 | Were outcome assessors adequately blinded to assess the primary outcomes? |  |  |  | If participants completed self-reported study forms, this item becomes N/A. |
| 8.1 | If outcome assessors were not adequately blinded, were specific methods used to avoid ascertainment bias (systematic differences in outcome assessment)? |  |  |  | Did the investigator describe possible and study-specific methods to avoid assessor bias? |
| 9 | Was the follow-up schedule the same in each group? (parallel design) |  |  |  | Did participants in each group follow the same timeline or were there variations? |
| 10 | Were the main outcomes analyzed according to the intention-to-treat principle? |  |  |  | Intention-to-treat analysis should include everyone who was randomized into the study, no matter if they completed the study or not. |
